# Redesigned Electrodes for Improved Intraoperative Nerve Conduction Studies during the Treatment of Peripheral Nerve Injuries

**DOI:** 10.3390/healthcare12131269

**Published:** 2024-06-26

**Authors:** Nathaniel Riemann, Jack Coursen, Laura Elena Porras, Bryan Sabogal, Xin-Hua Liang, Christian Guaraca, Allan Belzberg, Matthias Ringkamp, Gang Wu, Lily Zhu, Samantha Weed, Constanza Miranda

**Affiliations:** 1 Department of Biomedical Engineering, Johns Hopkins University, Baltimore, MD 21218, USAconstanzamiranda@jhu.edu (C.M.); 2 Department of Neurosurgery, School of Medicine, Johns Hopkins University, Baltimore, MD 21287, USA

**Keywords:** peripheral nerve, nerve conduction study, electrode, intraoperative neuromonitoring, action potential, signal artifact, nerve trauma injury, neurosurgical treatment, design process, clinical need

## Abstract

Traumatic peripheral nerve injuries (PNI), present with symptoms ranging from pain to loss of motor and sensory function. Difficulties in intraoperative visual assessment of nerve functional status necessitate intraoperative nerve conduction studies (INCSs) by neurosurgeons and neurologists to determine the presence of functioning axons in the zone of a PNI. This process, also referred to as nerve “inching”, uses a set of stimulating and recording electrode hooks to lift the injured nerve from the surrounding surgical field and to determine whether an electrical stimulus can travel through the zone of injury. However, confounding electrical signal artifacts can arise from the current workflow and electrode design, particularly from the mandatory lifting of the nerve, complicating the definitive assessment of nerve function and neurosurgical treatment decision-making. The objective of this study is to describe the design process and verification testing of our group’s newly designed stimulating and recording electrodes that do not require the lifting or displacement of the injured nerve during INCSs. Ergonomic in vivo analysis of the device within a porcine model demonstrated successful intraoperative manipulation of the device, while quantitative nerve action potential (NAP) signal analysis with an ex vivo simulated “inching” procedure on healthy non-human primate nerve tissue demonstrated excellent reproducible recorded NAP fidelity and the absence of NAP signal artifacts at all points of recording. Lastly, electrode pullout force testing determined maximum forces of 0.43 N, 1.57 N, and 3.61 N required to remove the device from 2 mm, 5 mm, and 1 cm nerve models, respectively, which are well within established thresholds for nerve safety. These results suggest that these new electrodes can safely and successfully perform accurate PNI assessment without the presence of artifacts, with the potential to improve the INCS standard of care while remaining compatible with currently used neurosurgical technology, infrastructure, and clinical workflows.

## 1. Introduction

Peripheral nerve injuries (PNIs) affect 20 million Americans, disproportionately affecting young individuals subject to motor vehicle accidents, falls, and industrial accidents [1,2]. The clinical presentation of PNIs varies from symptoms of mild discomfort to pain and loss of function that limits daily activities [3]. Muscles without nerve reinnervation typically exhibit irreversible deterioration after 12 to 18 months; therefore, PNI treatments are limited by the time necessary to reinnervate the muscle connected to the injured nerve [3]. As axons regenerate at a rate of about one inch per month, an injury further from the site of nerve–muscle innervation will take longer to fully regenerate, thereby shortening treatment time and requiring earlier clinical decision-making and intervention [3].

**Figure 1:**
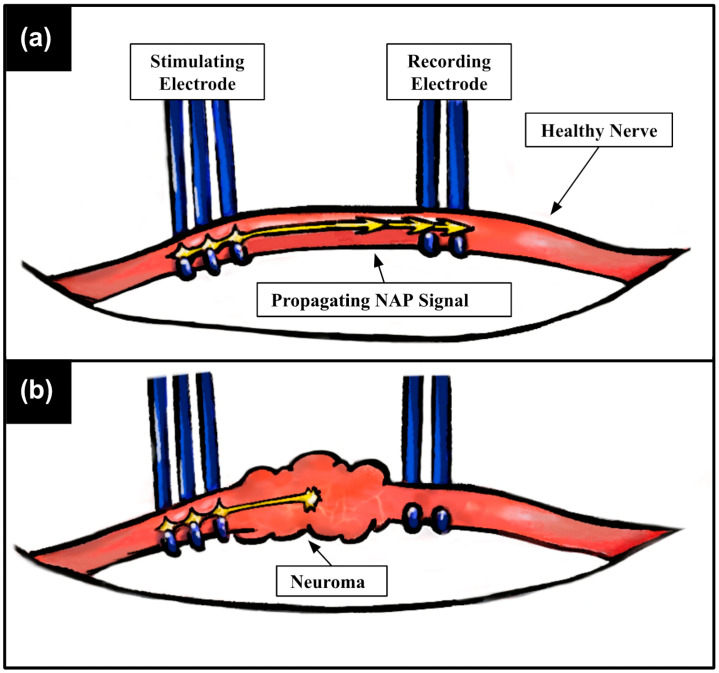
Current setup and method of peripheral INCSs. The injured nerve is lifted from the surrounding surgical field using a pair of standard intraoperative neuromonitoring (IONM) electrodes. (**a**) A tripolar stimulating electrode is used to induce nerve action potential (NAP) propagation down the nerve, which is later recorded by the bipolar recording electrode. A healthy nerve exhibits continuity of signal propagation, which is determined by the presence of a recorded NAP. (**b**) In a damaged nerve, the axons within the nerve sheath lose the ability to continuously propagate NAPs to the recording electrode, and for this reason, no signal is recorded. This stimulating and recording procedure is repeated as the stimulating electrode is incrementally moved across the site of nerve injury.

Once a nerve injury is identified, surgeons wait for a period of 3 to 6 months to provide time for potential axon self-regeneration, after which the nerve is reassessed using intraoperative nerve conduction studies (INCSs) to determine whether the nerve axons are successfully self-regenerating (requiring no further surgical intervention) or whether further surgical intervention is required to reinnervate the muscle with functional nerves [3]. During INCSs, the neurosurgeon first dissects into the surgical field until the injured nerve is identified and isolated. Next, the continuity of nerve action potential (NAP) propagation is assessed along the site of nerve injury using a tripolar stimulating electrode and bipolar recording electrode (Figure 1). During this procedure (also referred to as nerve “inching”), the neurosurgeon places the electrodes on each side of the predicted zone of injury, and an electrical stimulus is sent via the stimulating electrode. If a stimulated NAP successfully propagates through the injured nerve region and is recorded by the bipolar electrode (Figure 1a), successful nerve function is confirmed; conversely, the absence of a recorded signal (Figure 1b) indicates nerve discontinuity (lack of axonal function) through the injury, requiring further surgical treatment (e.g., direct nerve repair and autologous nerve grafting—current gold-standard treatments) [4,5,6,7,8]. Following this single assessment, the nerve inching procedure is continued by incrementally moving the stimulating electrode closer towards the recording electrode and repeating the aforementioned procedure at these subsequent electrode positions to precisely define the zone of injury, guiding the neurosurgeon’s clinical decision-making [9].

INCSs are a key and standard electrophysiological technique used to determine internal nerve axonal functionality. Other methods, including advanced functional imaging technologies, do not have the resolution necessary to assess internal axonal structure, and EMG only provides general information regarding nerve function at the late stage when muscle reinnervation is expected to occur [10,11]. Current INCSs are problematic due to electrical artifacts distorting the NAP recording, making definitive nerve assessment difficult [8]. Inching is sensitive to various disruptions:

- Nerve Lifting: Gently lifting the nerve via the electrodes will remove the electric ground from the electrophysiological nerve system, which disrupts the recorded signal by introducing signal artifacts in the NAP recording [12].
- Electrode Spacing: The spacing between the signal artifact and NAP on the recorded signal is proportional to the distance between the recording and stimulating electrodes on the nerve. Distances less than 4 cm between the electrodes can cause the NAP recording to be masked by a signal artifact; distances too long between the electrodes may allow the signal to interact with extrinsic factors [4].
- Isolation of the Electrode-Contact Sites: The currently used electrodes must isolate the nerve through gentle lifting and suspension from the surgical field, distancing the sites of stimulation and recording from surrounding tissues that may produce a misleading NAP signal. This may result from “spread”, where the signal propagates through surrounding muscle tissue instead of the nerve [4,11].
- Nerve–Electrode Contact: The prongs of the stimulating and recording electrodes must have proper contact with the nerve for stimulation and NAP recording. This includes minimizing contact with surrounding fluids in the body, as these mediums can disrupt the NAP [4].

The combination of a limited time frame for successful PNI treatment and nerve assessment inaccuracies can be detrimental to clinical outcomes. False-positive NAPs can result in a lack of surgical intervention in a non-regenerating axon, and false-negative NAPs can result in unnecessary surgery on nerves that would have regenerated on their own; both of these scenarios would restart the treatment process, providing less time for further intervention and full nerve regeneration before the innervated muscle exhibits irreversible atrophy. When patients wait to seek medical attention after injury or when an iatrogenic PNI is sustained through a different operation and the clinician waits to assess the development and extent of the injury, avoiding any further delays to muscle reinnervation may be critical to final functional outcomes [3,10,11].

To perform this nerve “inching” procedure, the current standard of care involves a set of standard IONM hook electrodes. The length of each electrode is electrically insulated, while the hook structure is fully uninsulated; this requires the neurosurgeon to lift the nerve up from the surrounding surgical field to (1) maximize the sufficient quality of electrode contact with the nerve and (2) to ensure that electrical stimulation and recording are limited exclusively to the nerve (as opposed to any surrounding tissue). Unfortunately, this workflow also exposes the nerve to additional strain via nerve lifting.

Through a structured investigation of background research, clinical observation, and a structured design process, our team identified the existence of a clinical need: PNI patients, neurosurgeons, and their operating team require a more accurate and consistent method of determining the presence or absence of functioning axons in a zone of injury to guide further treatment decisions. The objectives of our group were to implement a rigorous design process to create new stimulating and recording IONM electrodes that do not require the displacement/lifting of the nerve. Our group developed, fabricated, and verified our novel electrode design in terms of ergonomics, signal accuracy, and safety of implementation utilizing in vivo, ex vivo, and stimulated nerve experimental setups, respectively.

## 2. Materials and Methods

Section 2 describes the design process that our interdisciplinary team of biomedical engineers and end-users went through to develop our novel IONM electrode design, as proposed by Miranda et al. 2020 to increase the likelihood of final device adoption [13,14].

### 2.1. Identifying the Clinical Need and Establishing Design Requirements

We were first approached by scientists and a neurosurgeon from the Neurosurgery Department at a prominent research institution’s hospital who needed a better way to assess nerve function in the setting of PNI and to assess the need for surgical intervention. After an extensive literature search across journals of clinical and surgical medicine, biomedical research, and national biostatistics reports regarding the physiopathology of peripheral nerve injuries, we engaged in applied ethnographic methods [13,14], such as participant and non-participant observation in the field [15,16] and open-ended interviews [17,18].

Our sample of participants consisted of 15+ neurosurgeons, neurologists, physician’s assistants, electrophysiologists, and additional operating team staff (i.e., circulating nurses, technicians, anesthesiologists, residents, and medical school students) with whom we observed the neurosurgical workflow across a variety of neurosurgical procedures (e.g., nerve tumor removal, nerve decompression surgery, autologous nerve grafting treatment of acute peripheral nerve trauma), including pre-operative clinic visits, patient consent, anesthesia, peripheral nerve assessment, and the ultimate neurosurgical treatment. Our firsthand observations into the technical and ergonomic challenges of peripheral nerve assessment included induced nerve tension from the hook electrodes, ergonomic inefficiency (two surgeons were required to perform the two electrode nerve assessments), and ambiguity of recorded nerve action potential signals due to electrical artifacts that resultantly complicated and delayed surgical treatment decision-making. Further collaboration with electrophysiologists provided insight into the bioelectrical basis of observed clinical pinch-points, how to mitigate emergent signal artifacts, and improved ergonomics within the clinical workflow.

All of this information was collected and interpreted in our Design History File, where our team documented all steps undertaken in the development of our medical device and in synthesizing actionable design requirements (Table 1) for our novel solution via converging ideation by the team [19,20,21,22].

### 2.2. Prototyping and Fabrication of the Device

Addressing DR02, we ideated and vetted several electrode configurations, settling on a flat “sandwich” or chopstick-style clamp electrode to fasten around the nerve from above to facilitate disengagement in the event of emergency electrode removal. The electrode leads were electrically insulated aside from precise contact areas designated for nerve contact only, thereby isolating NAP stimulation and recording to only the nerve itself. We designed a slider component to be controlled by one’s index finger, thereby providing a neurosurgeon with fine motor control over the exerted forces on the nerve by the electrode and the expanding/retracting fastening mechanism of the device (DR04, DR06). The electrode leads and slider mechanism were within an original handle structure designed to support the fastening mechanism. The initial iterative prototyping stage of the design process was facilitated using 3D printing software and printers to quickly produce the electrode slider and housing components.

**Figure 2:**
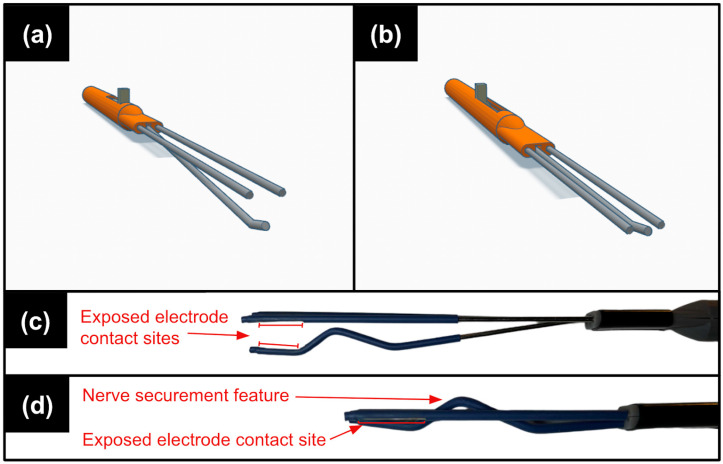
Electrode design prototypes. (**a**) Computer-aided design (CAD) render of the electrode prototype used in in vivo preliminary ergonomic analysis (see Figure 3) with uninsulated leads fully extended. Electrode leads extend 9 cm from the handle, with a 1.5 cm spacing between the top two parallel leads and bottom lead. The handle is 15.5 cm in length and 1 cm in diameter. (**b**) CAD render of aforementioned electrode prototype with the slider and leads fully retracted. Electrode leads extend 6 cm outside the handle in this position. (**c**) Side view image: fully extended stimulating electrode leads of the finalized prototype. (Note: the electrode handle, slider components, and lead extension lengths remain unchanged from the previous iteration.) (**d**) Side view image: fully retracted electrode leads of the aforementioned electrode. This iteration contains a nerve securement “hump” feature that prevents the nerve from sliding backwards into the device. Note that the recording electrode is dimensionally identical to the stimulating electrode but uses a fully insulated middle electrode lead for physical support only and has no electrical activity.

The overall finalized design (Figure 2) comprised four primary structural components: a “pencil grip”-style handle, an index finger-adjusted linear sliding mechanism to open and close the electrode clamp around the nerve, embedded stainless steel electrode leads for nerve stimulation and recording, and polyolefin electrode insulation around each electrode lead, with cut-outs for necessary sites of stimulation and recording.

Many dimensions and materials of the electrode components were chosen to be similar to the currently adopted IONM Cadwell Disposable 180° Degree Double Hook (Cadwell Industries Inc., Kennewick, WA, USA), with 1.5 mm diameter stainless steel electrode leads, a fully extended length of 9 cm outside the handle, and 5 mm spacing between recording electrode leads [12]. The prototype fabrication process was as follows: The electrode handle/housing and slider component were printed using a PLA 3D printer (with later iterations using a resin 3D printer). Next, the stainless steel electrode leads were manually cut to the desired length and shaped with pliers to their desired form (such that the middle electrode lead would expand downwards as the electrode was expanded to a maximum width of 1.5 cm). The electrode leads were glued into the 3D-printed slider component, after which polyolefin insulation was applied to each lead using a heat gun. Flexible 17-gauge tin-plated copper wires were soldered to the back of the electrode leads—on the recording electrode, individual wires were soldered to the right- and left-most leads, while the middle lead was fully insulated; on the recording electrode, the right- and left-most leads were soldered to one wire, while the middle lead was soldered to a separate wire. Electrode-nerve contact areas were manually cut out of the insulation (1 mm by 6 mm) using a hobby knife. Lastly, the electrode lead and slider assembly were sealed within the electrode handle/housing.

**Figure 3:**
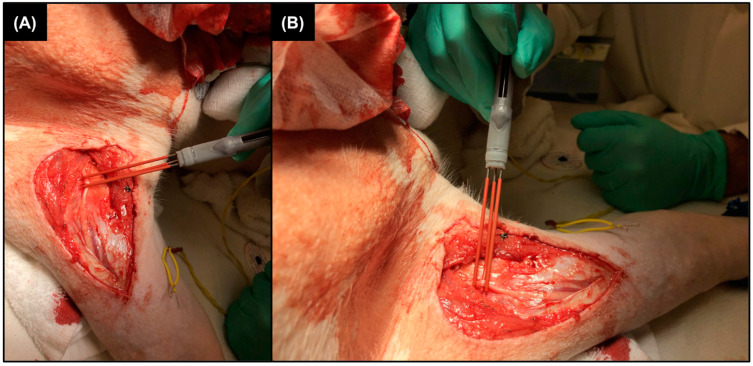
Preliminary ergonomic analysis within an animal model. (**A**) Aerial view of the electrode prototype secured on the ulnar nerve of a pig; the surgeon retracted the slider mechanism until the electrode device was safely secured onto the nerve. (**B**) Frontal view of electrode securement on the ulnar nerve.

Upon completion of an initial functional prototype of the electrode design (represented by Figure 2a,b but with polyolefin lead insulation), our electrophysiologist and neurosurgeon clinical mentors performed a preliminary ergonomic analysis of the device within an in vivo porcine model (Figure 3); the animal had been recently euthanized following the end of a different experimental study. (This animal study protocol PR21M249 was officially approved by the Animal Care and Use Committee of the Johns Hopkins University on 09/14/21.) Using the electrode sliding mechanism, the leads of a single electrode were gently secured by the neurosurgeon onto an exposed, non-injured ulnar nerve; no electrical stimulation or recording was performed, as indicated by the visible yellow disconnected electrode wires. Following this preliminary analysis, a final iteration of electrode design was developed (Figure 2b,c) to further improve the securement of the nerve on the points of the exposed electrode lead contacts.

### 2.3. Ex Vivo Verification Testing

**Figure 4:**
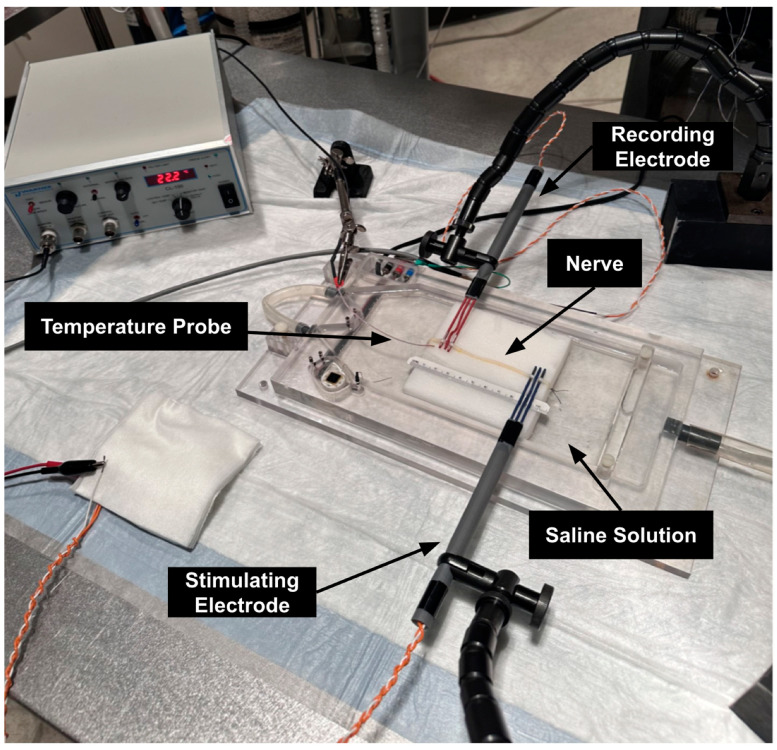
Ex vivo experimental setup. Note that the standard “normal” stimulation convention was with the middle electrode lead as negative and the left- and right-most leads as positive; this was then flipped during the reversed stimulation polarity. Both electrodes were secured onto the nerve in such a way that the nerve was not lifted.

Upon completed design and assembly of the finalized electrode prototype that addressed our aforementioned needs criteria, our team performed ex vivo verification testing on a healthy (i.e., non-injured) monkey tibial nerve to ensure that the devices could successfully stimulate and record NAPs without the presence of signal artifacts. (This animal study protocol SW23M55 was officially approved by the Animal Care and Use Committee of the Johns Hopkins University on 03/17/23.) The tibial nerve was placed on a 10 by 10 cm square of gauze (representing the surrounding surgical tissue within an in vivo surgical field) immersed in a normal saline (synthetic interstitial fluid) solution bath. The experimental setup is shown in Figure 4. A simulated nerve “inching” procedure was performed, using the following procedure: First, the stimulating and recording electrodes were secured onto the nerve and placed 66 mm apart from one another; this was considered the first of six electrode positions of the inching procedure. At each position, a 4 mA, 0.05 ms pulse width constant current stimulus was delivered every 5 s (0.2 Hz) via the stimulating electrode, from which a resulting NAP was recorded via the recording electrode. Following this, the recording electrode remained stationary, while the stimulating electrode was “inched” into the next position, moving 10 mm closer towards the recording electrode. (I.e., the electrodes were 56 mm apart in position two, 46 mm apart in position 3, etc.) Saline solution was periodically applied to the nerve to preserve the physiological properties of the setup. This procedure was repeated through the sixth electrode position, at which the electrodes were placed 16 mm apart. At this final position, the electrical stimulus polarity was reversed to further investigate the presence or absence of signal artifacts.

### 2.4. Electrode Pullout Force Testing

Following ex vivo verification testing, the electrodes were tested to determine the force needed for emergency device removal from the nerve by a surgeon. Three-dimensionally -printed molds were used with Smooth-Sil 940 silicone to form three nerve models approximately 7 cm in length and of varying diameters: 2 mm, 5 mm, and 1 cm.

**Figure 5:**
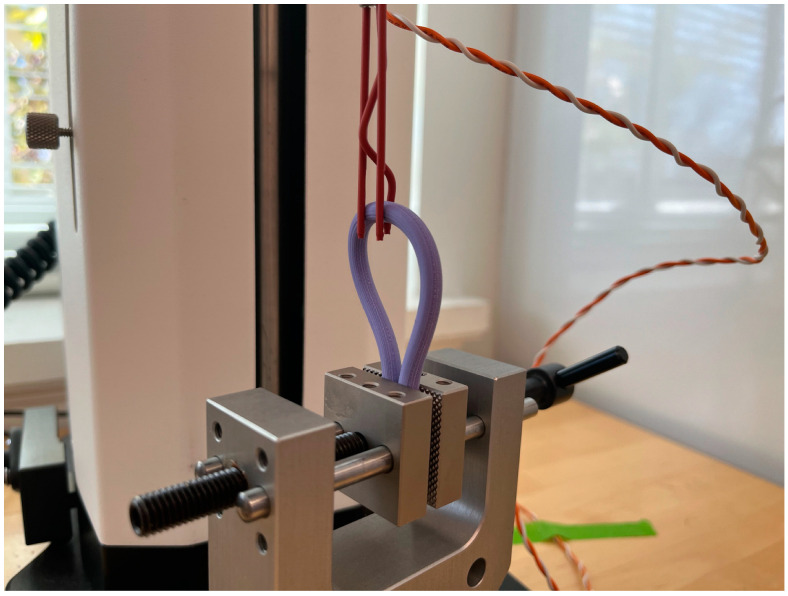
Experimental setup for electrode pullout force testing on a 5 mm diameter nerve model. The electrode leads were fully retracted back for a standardized maximum securement on the nerve model for each testing trial; a 5 mm nerve model is shown here; 2 mm and 1 cm nerve models were tested as well.

An ESM303 Mark-10 Motorized Test Stand and Model M5-20 Force Gauge (Mark-10 Corporation, Copiague, NY, USA) were used to measure the force required to remove the electrode from the nerve model. First, the nerve model was fixed/clamped to a stage at the base of the Mark-10; next, the electrode was similarly clamped to the movable strain-gauge component of the sensor. Upon securement of the electrode and nerve model, the electrode was fastened and fully retracted onto the nerve model. Under the default settings of the Mark-10, the movable strain gauge component was adjusted to the initial position (Figure 5), and the force gauge was calibrated, from which the electrode was incrementally pulled upwards at a constant rate with linear movement. This was stopped once the electrode detached from the nerve model. The plots and data tables for each run were saved, and this procedure was performed for *n* = 6, *n* = 6, and *n* = 8 trials for the 2 mm, 5 mm, and 1 cm diameter nerve models, respectively.

## 3. Results

Successful completion of our final design yielded a device which directly satisfied all of our articulated design requirements (Table 1). With respect to maximizing the ease of adoptability and minimizing change/disruption to the current surgical workflow (DR01), we designed the device to remain compatible with existing signal reading and stimulation infrastructure while maintaining the critical dimensions and two-electrode setup of the currently used tripolar stimulating and bipolar recording IONM hook electrodes. As a result, the integration of these devices into the current surgical workflow should only require replacing the current IONM electrodes with our new electrode.

**Figure 6:**
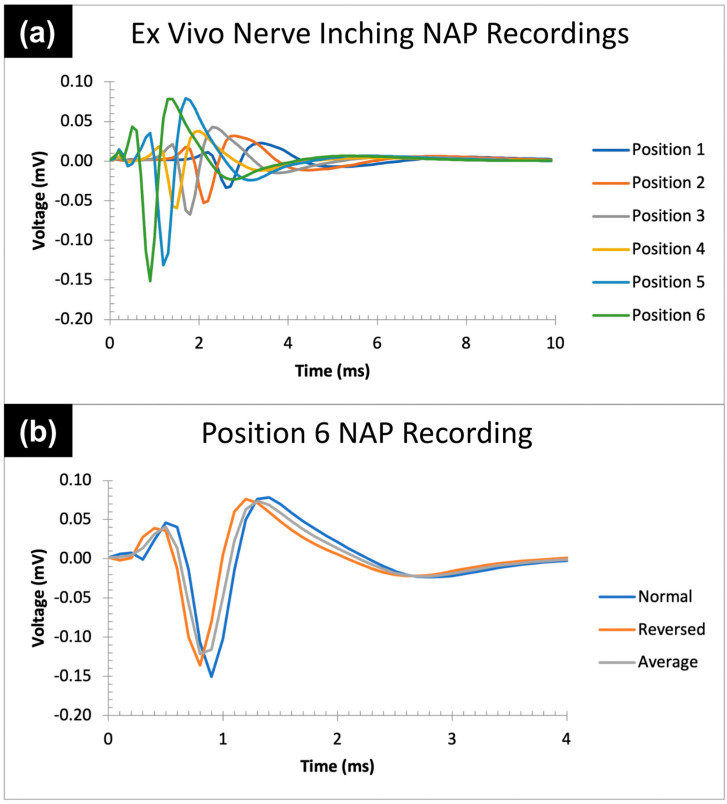
Recorded ex vivo NAP signals throughout the nerve inching procedure. (**a**) Recorded NAP signals for each of the six electrode inching positions. (**b**) Recorded NAP at the sixth and final electrode position produced by normal (blue) and reversed polarity (orange) electrical stimulations; an average of the two is shown as well (gray).

Regarding the mitigation of nerve lifting and surgical perturbations (DR02), our electrodes are designed to easily secure around an individual nerve resting within the surgical field rather than requiring lifting force to pull the nerve up and away. This “sandwich” or “chopstick”-like securement onto the nerve allows vertical surgical-accessible, nerve-specific securement while the nerve rests in place, thereby allowing NAP stimulation and recording while minimizing nerve lifting and perturbation within the surgical field (as demonstrated by the preliminary in vivo ergonomic analysis shown in Figure 3). We demonstrated minimal perturbations of the nerve and surgical field as a method of recorded artifact mitigation/prevention, as shown in published academic literature and our collected ex vivo data (Figure 6), as well as a step further towards patient safety, as current methods of peripheral nerve surgery can commonly cause nerve scar tissue formation [11,23].

This newly designed fastening mechanism is precisely engineered to extend and retract along a continuous range of securement sizes (DR03)—from a smaller nerve diameter of 1–3 mm to large (e.g., sciatic) nerve diameters of over 1 cm—allowing the device to accommodate various peripheral nerve diameters in a ‘one-size-fits-all’ manner (Figure 2) [24]. The extension and retraction of our variable-diameter fastening mechanism is operated by a sliding element integrated into the electrode handle; operation of this mechanism with the lateral side of the surgeon’s index finger while the device is held with the thumb, similar to the grip of a pen or pencil, provides fine motor control and thereby precise control of the forces exerted on the nerve by the surgeon/electrode (DR04), as seen in Figure 3 [25]. Furthermore, full use of the device is accomplished using only one hand, thereby allowing simultaneous manipulation of a tripolar stimulating and bipolar recording electrode during the peripheral nerve assessment procedure by a single surgeon, eliminating the need for two operating surgeons for IOMN assessment (DR06).

Regarding the ex vivo data collection, six NAP signals were recorded from the six total electrode inching positions to verify successful electrode stimulation and recording function, as well as to verify recorded signal quality without artifact presence. Additionally, an extra NAP from a polarity-switched stimulus in the sixth (final) electrode position was recorded with the intention of investigating artifact presence/absence. The recorded waveforms are shown in Figure 6.

As the electrodes were inched closer together, the NAP amplitude increased from a depolarized peak of −0.034 mV in position 1 to a depolarized peak of −0.15 mV in position 6, and the NAP latency shortened from approximately 1.7 ms in position 1 to approximately 0.1 ms in position 6. This is bioelectrically justified, as bringing the point of electrical current stimulation closer to the site of NAP recording should yield a faster presence of NAP signal at the recording electrode, as well as a higher measured potential. Additionally, repositioning of the stimulating electrode throughout the inching procedure did not at all affect or compromise the quality of NAP recordings. Even when the stimulating electrode was brought the closest to the recording electrode, the recorded NAP remained consistent, without any trace of nerve-lifting artifacts or electrical stimulus artifacts (i.e., when the site of stimulation is brought too close), as shown by the consistent NAP signal from normal- and reverse-polarity stimulations in Figure 6b.

**Figure 7:**
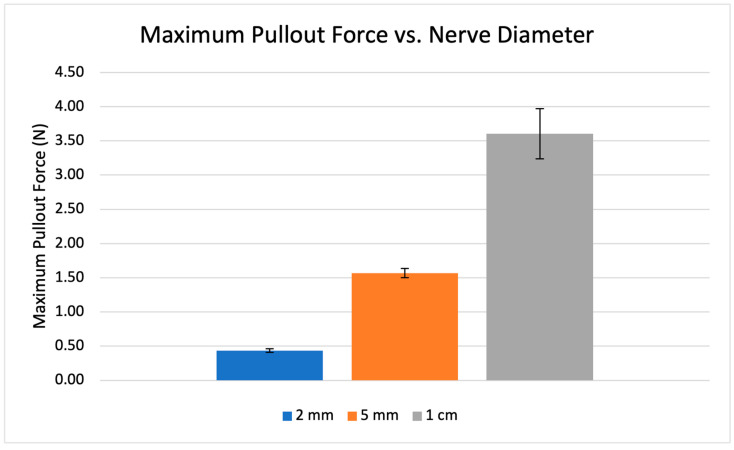
Recorded maximum pullout forces required for electrode removal from nerve models of three different diameters. Error bars represent standard error. Statistically significant differences in average pullout forces between the three groups (*p* < 0.001) were calculated using a two-tailed, unpaired *t*-test.

With respect to the electrode pullout force testing, the average required forces to remove the electrode from the 2 mm, 5 mm, and 1 cm nerve models were 0.43 N, 1.57 N, and 3.61 N, respectively (Figure 7).

## 4. Discussion

The ex vivo nerve inching procedure verification testing quantitatively verified the stimulation and recording functionalities of our electrodes. As shown in Figure 6a, each of the six NAP recordings throughout the nerve inching procedure yielded clean signals without the presence of artifacts, due to the non-lifting method of electrode engagement with the nerve. The data also demonstrate that the repositioning of the electrodes across different positions during an “inching” procedure does not affect or compromise the recorded signal quality, showing a robustness in accurate signal recording throughout the electrode manipulation within the surgical environment. The absence of the recorded signal artifact is further shown in Figure 6b, showing consistency in the recorded NAP signal from a normal vs. reverse polarity of electrical stimulation, even when the stimulating electrode is brought closest to the recording electrode.

In the case of a clinical emergency in which the electrodes are quickly removed from the nerve—or if the electrodes/wires are pulled by accident—it is important that minimal force be required to disengage our electrodes from the nerve to avoid further nerve injury. Our collected pullout force testing demonstrates that this is the case with the newly designed electrodes via a quantitative maximum pullout force experiment across different nerve diameters, similar to the investigation performed by Lamadé et al. 2011 (which cites a maximum nerve tensile force of 21 N/mm^2^ of the median nerve) [26]. Our collected results (Figure 7) were then compared to the data published by Rickett et al., in which the NAP amplitude remained statistically unchanged at a 5% strain but diminished at a 10% strain [27]. This suggests that a minimum threshold for stretched nerve damage on NAP amplitude occurs between these two values, which corresponds to calculated stress forces of 1.1 to 2.5 N/mm^2^, respectively, incorporating the average 1.135 mm^2^ guinea pig cross-sectional nerve area from the study. The calculated stress forces of our electrode (0.03, 0.02, and 0.01 N/mm^2^ for the 2 mm, 5 mm, 1 cm diameter nerve models, respectively) lie well within the published safety guinea pig nerve threshold and further suggests that this will remain the case for larger human nerves. It is worth noting that this maximum pullout force was measured with the electrodes exerting their maximum securement at a full lead retraction onto the nerve model; in reality, the nerve can be secured with less than full retraction, thereby requiring even less force for emergency removal.

Alongside these benefits demonstrated by the verification of our design criteria in the final electrode design (Table 1; see Section 3), there still currently remain limitations in our assessment of the device. Namely, functional testing is still required to fully verify ergonomic electrode manipulation during simultaneous successful electrical stimulation and NAP recording within an in vivo surgical field, instead of verifying these design criteria separately (and partially within an ex vivo setup), as shown in this study. Additionally, the reproducibility of simultaneous ergonomic and functional electrode verifications within an in vivo setting should be shown across different anatomical sites of surgery, demonstrating that the electrodes can robustly function across intraoperative settings. Because our electrodes are manipulated via fine motor control, this requires neurosurgeons to actively perform precise surgical technique in order to ensure IONM success and maximize patient safety; this may likely require a degree of training/familiarization with this new instrument.

## 5. Conclusions

This study introduces a novel IONM electrode design to improve intraoperative NAP recording during the treatment of PNIs, in which current electrodes perturb the surgical environment, resulting in signal artifacts and potential iatrogenic nerve damage from surgical lifting. In collaboration with a team of electrophysiologists, neurosurgeons, and neurologists, our team articulated this clinical need and developed a set of design requirements to guide the development of redesigned stimulating and recording electrodes. Our redesign incorporated improved ergonomic design factors to ensure ease of surgical use and successful electrode contact across all diameters of nerves while allowing nerve contact with minimal nerve and surgical field perturbation/displacement; this ultimately addressed every ideated design requirement. Quantitative verification testing demonstrated the proper electrical function and ergonomic device manipulation on nerve tissue and an excellent safety profile with pullout force testing well within established safety limits. Ultimately, this innovation has the potential to improve the standard of care in peripheral nerve assessment, providing neurosurgeons and their operating teams with a valuable tool when making critical surgical decisions.

## Figures and Tables

**Table 1:** Design criteria and corresponding design affordances regarding electrode ideation and development. The six design requirements (left column) represent a comprehensive set of considerations by our group when ideating a new solution concept. The corresponding design affordances (right column) articulate the means by which each design criterion will be addressed in the ideated solution.

Design Requirement	Corresponding Design Affordance
DR01. Minimize invasiveness/disruption to the surgeon’s workflow	The principle of the instrument is kept in line with older solutions. The neurosurgeon should be capable of using our new device without substantial new training, thereby streamlining clinical adoptability.
DR02. Minimize/eliminate nerve lifting and perturbation to the surgical field	Electrode placement/securement should not require lifting of the nerve and should minimally perturb the surrounding surgical field, both mechanically and electrically. This will both minimize the physical risk to the wounded area and improve recorded signal quality by the surgeon.
DR03. Accounting for variable nerve diameters	The differences in physiology between patients and within patients at different points of the body require a device that accounts for the variability in nerve diameters. This design will improve the safety of the device by ensuring that safe compressive forces are exerted on the nerve. This newly designed electrode will also improve upon the adaptability of the device by reducing decision-making in the workflow when choosing which electrode size to use for the nerve at hand.
DR04. Ergonomic use of device control using a single hand	To accommodate a set of two electrodes used during peripheral nerve assessment, as well as the added functionality of the new device, each electrode should be ergonomically operable with a single hand.
DR05. Sufficient electrical contact between nerve and electrode.	Optimal, reproducible quality of nerve stimulation and NAP recording should be accomplished via secure, consistent physical contact between the electrodes and the assessed nerve; this can be indicated by the metric of constant contact surface impedance of this interface.
DR06. Direct control of forces exerted on the nerve via fine motor control.	In the setting of sufficient electrode securement for adequate nerve stimulation and NAP recording, our solution should provide surgeons with extremely fine control of the forces exerted on the nerve by the electrodes, i.e., via fine motor control.

## Data Availability

Data supporting the results are stored on the personal computers of co-authors Nathaniel Riemann, Matthias Ringkamp, and Gang Wu and are backed up on a personal Google Drive. The data are available upon request.
